# From conflict to partnership: growing collaboration between police and NGOs in countries with concentrated epidemics among key populations

**DOI:** 10.7448/IAS.19.4.20939

**Published:** 2016-07-18

**Authors:** Nicholas Thomson, Diane Riley, Anne Bergenstrom, Jenae Carpenter, Alex Zelitchenko

**Affiliations:** 1 Johns Hopkins Bloomberg School of Public Health, Baltimore, USA; 2 University of Melbourne, Melbourne, Australia; 3 Canadian Foundation for Drug Policy, Ottawa, Canada; 4 United Nations Office of Drugs and Crime, Islamabad, Pakistan; 5 University of Melbourne, Melbourne, Australia; 6 Law Enforcement and HIV Network, Bishkek, Kyrgyzstan

**Keywords:** HIV, key populations, police, NGOs, partnerships, reform, collaboration, donors, resourcing, roadmap

## Abstract

**Introduction:**

Between September 2012 and December 2015, a series of national and regional consultations, aimed at resolving a persistent dynamic of conflict between law enforcement agencies (LEAs) and civil society organizations (CSOs) working on issues of access to HIV services in high-priority countries for people who use drugs have been organized by the HIV/AIDS Section of the United Nations Office on Drugs and Crime, the Joint United Nations Programme on HIV/AIDS, the Law Enforcement and HIV Network (LEAHN) and other international organizations. The aim of these consultations has been to understand, at a national and regional level, the key points of tension between police and CSOs and how to overcome these tensions to enhance access to and uptake of services by key populations, including people who inject drugs, sex workers, men who have sex with men and transgenders. This commentary briefly describes the methods, process, content and key outcomes of these consultations held across diverse number of countries and regions, including Africa, South East Asia, South Asia, Central Asia, Eastern Europe and Latin America.

**Discussion:**

While the context varies, this paper highlights that there are commonalities that drive a persistent dynamic of conflict and therefore also common methods for resolution of conflict and forging partnerships. Both policing and CSOs have key sectoral responsibilities and reform agendas to implement to ensure that as an individual agency they are able to meet their obligations as partners in the HIV response. Using the key outcomes of discussions and recommendations from these consultations and drawing on existing literature, the objective of this paper is to present a preliminary model that roadmaps the critical path from resolution of conflict to partnership between LEAs and CSOs.

**Conclusions:**

This paper seeks to highlight that critical resources are required to support ongoing development and harnessing of partnerships between LEAs and CSOs and argues that these resources should not just come from global HIV funding mechanisms but should be part of a more mainstreamed security sector reform agenda that understands the mutual benefits that programming for human rights–based policing reform would have on HIV, development and security.

## Introduction

In the global fast track pursuit towards ending HIV by 2030, universal access to combination HIV prevention and treatment services, and an end to discrimination, are considered critical components [1]. Pharmacokinetic advances and evidence of the efficacy of pre-exposure prophylaxis on HIV prevention with key populations [2,3] make it possible to envision a world where HIV is no longer a global public health threat. However, concentrated epidemics of HIV persist in many countries, specifically among key populations including people who inject drugs [4], men who have sex with men [5], sex workers [6] and transgenders [7]. The inability to foster a truly enabling environment where key populations have universal access to combination HIV services at scale remains a significant reason why we have not been able to reverse many concentrated epidemics.

Legal and policy environments that either criminalize the behaviour, or the person engaging in the behaviour, are widely documented to be significant barriers to efforts aimed at reducing HIV incidence in concentrated epidemics [8]. In addition, there is a vast body of literature describing the negative impact that some police practices can have on both risk behaviour and access to and uptake of services for key populations. Studies among key populations continue to document that the fear of arrest [9], physical intimidation and violence at the hands of the police [10], frequency and threat of police raids [11] and police bribery [12] are variously associated with the sharing of needles [13], decreased access to methadone maintenance treatment [14], decreased condom use [15] and decreased access to (or the cessation of) anti-retroviral therapy [16].

Research has also sought to understand the perspectives of police at the interface between policing and HIV programmes working with key populations and have variously described contributors to negative policing behaviour including the poor understanding of HIV and HIV programmes, the lack of appropriate police training, poor communication from HIV programmes to police [17] and structural drivers of poor police performance such as low salaries and the setting of arrest quotas that specifically require police to target people who use drugs [18]. In response to the ongoing tensions between police, HIV programmes and the people that need access to these programs, various multilateral agencies [19], researchers [20] and civil society organizations (CSOs) [21] have recommended the need for enhanced partnerships between police and HIV programmes.

The benefits of police and HIV programme partnerships have also been variously described and while the literature is in its infancy, these efforts have highlighted that partnership can lead not only to reductions in HIV-risk behaviour and increased access to services [22] but can also be associated with improvements in indicators of interest to police, including crime [23], perceptions of safety and community trust in policing.

Between September 2012 and December 2015, a series of national- and regional-level consultations, aimed at resolving a persistent dynamic of conflict between police and civil society–led HIV programmes working on issues of access to HIV services for key populations, were organized by the HIV/AIDS Section of the United Nations Office on Drugs and Crime (UNODC), the Joint United Nations Programme on HIV/AIDS (UNAIDS), the Law Enforcement and HIV Network (LEAHN) and other international organizations in high-priority countries. This effort resulted in two regional dialogues across Central Asia and Eastern Europe (2011, 2013), and national-level dialogues in Vietnam (2013), Myanmar (2013), Thailand (2013), the Philippines (2014), India (2013, 2014 and 2015), Pakistan (2013 and 2015), Kyrgyzstan, Tajikistan, Kazakhstan (2013) and Tanzania (2013). These workshops were facilitated by a combination of international trainers as well as national law enforcement experts. The aim of these consultations was to understand, at a sub-national, national and regional level, the key points of tension between police and programmes and how these tensions can be overcome to enhance both service delivery for key populations and the community safety objectives of policing. The consultations were built around four facilitated sessions tailored specifically to the country or regional context and were designed to create a space for law enforcement agencies (LEAs) and CSOs and/or non-government organizations (NGOs) to share respective positions, concerns and ideas for enhancing future collaboration.

The opening session introduced the consultation and its objectives, the second session drew on the literature and global efforts that have explored the formation of partnerships between LEAs and specific attention given to topics such as the importance of leadership, police reform, CSO capacity building, mechanisms of formal and informal communication between sectors and the evaluation of partnership strength and success. The third session asked participants to consider what a two-year programme to build partnership may look like, including critical key components, a potential agenda of issues to work on, a timeline of milestone events, the key actors that would need to be involved and a potential monitoring and evaluation framework for assessing the efficacy of the partnership building exercise. The fourth session envisioned partnership and proposed potential steps, including a discussion on the development of an overarching national programme to enhance partnership between LEAs and CSOs.

Each workshop was evaluated using a standard pre- and post-evaluation questionnaire which captured information on knowledge, attitudes and beliefs of participants from both LEAs and CSOs. Consultation reports were also produced. The consultations employed simultaneous language translation where more than one language was being used. The objective of this commentary is to draw on an analysis of the consultations and the existing literature and propose a potential model that outlines a two-year roadmap for the resolution of conflict and the building of sustained partnership between LEAs and CSOs working on HIV programmes. This paper then proposes that efforts and resources aimed at creating an enabling environment should be mobilized with middle- to long-term timeframes so that partnerships between police and HIV programmes can be fostered and sustained and indeed evaluated for efficacy through both a public health perspective and a criminogenic lens.

## Discussion

**Figure 1:**
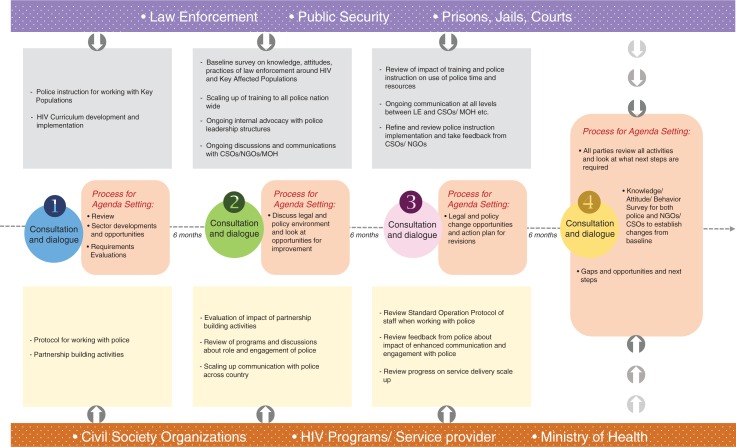
Model depicting two-year roadmap designed to enhance and sustain partnerships between key law enforcement and health stakeholders in response to concentrated epidemics among key populations.

The conceptual model describes the need for regular facilitated dialogue and the practical steps that each sector would need to take to ensure they are upholding their commitment to building partnerships (Figure 1). Given the fact that different stakeholders that exist across different contexts may have a significant role in shaping the partnership and ensuring applicability in different settings, this model expands the number of agencies considered to be principally LEAs and in addition expands the agencies working on HIV programming for key populations. The justification for certain component is outlined through presentation of some of the findings of the consultation and is supported by existing literature below.

### Related to policy, protocols and training

Participants from across LEAs in many of the national and regional consultations described a confusing policy environment for the provision of harm reduction and other HIV-related services for PWIDs. Whilst in some countries there was a stated supportive national government harm reduction policy, this did not necessarily translate into an specific stated or implemented policy, protocol or instruction for national police. Furthermore, many LEAs described a very limited understanding of harm reduction policy and practice among grassroots policing at the local community level. Further analysis reveals that in many countries, specific HIV and harm reduction training is entirely absent from police training curriculum or has only been piloted. The role of police training academies in cascading knowledge and skills to the lower levels of the police force was considered as critical by most participants. Reaching police who have already been through police academies with updated training was also mentioned as a distinct challenge in many countries. Finally, participants from LEAs recommended that development of a work place policy on HIV for LE officers should be considered where such is not available.

The conceptual model outlines the need for the development of standard operating protocols, specifically outlining how police can work and interact with key populations as a pre-requisite for police to be able to play a more positive role in supporting service access for key populations. While police protocols for working with key populations were almost completely absent from the majority of police institutes involved in the consultations, examples of the development of such protocols exist including in Cambodia [24] and Kyrgyzstan [25]. Protocol development is a low-cost intervention that should be considered in national programme design and supported by HIV donors.

Participants from CSOs in some countries described specific protocols that had been developed to guide how HIV programme workers, such as peer educators and outreach staff, should work with police, including outlining advocacy strategies, being formally credentialed and the need for regular communication between the programmes and local police. Protocols for HIV programmes to work with police were not uniformly developed or implemented across all countries which many participants from CSOs saw as a practical opportunity to increase their engagement and collaborative working with police. CSOs described many ongoing barriers to working relationships with police such as the use of urine testing as evidence of drug use, arbitrary arrest of members of key populations or HIV programme staff and the inability of programmes to identify the right level of authority to engage with in advocacy efforts.

### Related to programmes and community engagement

Participants from LEAs described some of the challenges related to the formation of partnership as the expectations of community on the role of police in relation to drug use. There was a widely held perception that the community at large was not completely aware of or on board with HIV-related programmes working with people who use drugs (PWUDs). In response to this, participants from CSOs challenged LEAs to work with them more directly in advocating and educating the community on the role of HIV programmes and indeed the role of police in supporting those programmes. There was recognition that the practical implementation of this strategy would require significant preparation and dialogue between LEAs and programme workers including senior-level police advocacy for the strategy. The notion that police should advocate and support harm reduction programmes has been described as being in line with the core principles of policing for public health [26].

LEAs in some countries described the need for evaluations of current programmes to be made more readily available and the need for programmes to adhere to some of the foundational principles of needle syringe programmes, including the need to ensure that needles were not discarded in public spaces, as that undermined the community sentiments towards the programmes and indeed put pressure on the police to crack down on the programmes. Participants from CSOs countered that there was indeed a need to also evaluate the role of police in engaging with programmes in response to any police training of advocacy efforts undertaken to assess the compliance with any implemented protocols.

### Related to partnership and sustainability

Participants from both LEAs and CSOs thought that a partnership between LEAs and CSOs is much needed and recommended additional consultations and dialogues to take place as a partnership between police and CSOs was considered a “win–win” situation with benefits to both sectors. Participants described a series of next steps that related to a range of individual sectoral responsibilities to lay the ground for sustained partnership. Participants from LEAs described the need for the development of training and curriculum materials and the need to incorporate these into the curricula of the police training academies. Where this was already implemented, participants recommended that police already working in communities should also receive training. Police training has been shown to have an impact on improved police knowledge and attitudes towards harm reduction programmes [27]. Investing in police training and education has also been shown to be scalable and effective at reducing HIV risk among key populations in limited settings [28].

Participants from CSOs discussed the need for HIV programmes to have a consistent approach to working in the field with police. The development of this consistent approach would require internal protocols, engagement strategies with police embedded in programme design and the early participation of LEAs when programmes are starting on the ground. Several participants also recommended that a national- or sub-national-level coordination committee or working group, consisting of representatives of LEAs, prison and health officials and CSOs be established to facilitate coordination and problem solving. A related recommendation was for a “nodal officer” to be identified at the sub-national level, as relevant, to facilitate coordination between LEAs and CSOs. The use of “nodal officers” has been employed as a strategy to increase support for HIV programmes in India [29].

Participants also described several collaborative opportunities that could be jointly organized including regular engagement focusing on mutual teaching, learning and situational analysis. The conceptual model is based on the notion that an ongoing facilitated dialogue between police and programs is a critical platform providing both sectors opportunities to work towards a proverbial “win-win” At the crux of communication and engagement between LEAs and CSOs is the need for the dialogue to be based on a set of principles that include transparency, fairness and respect which are guiding principles also outlined by the European Platform for Policing and Human Rights [30]. The potential for internships or secondments across sectors was discussed as was the need for joint agenda setting towards a sustained partnership effort. Critical to all of these suggestions was the need for the development of a monitoring and evaluation framework that would examine the progress of partnership from both LEAs and CSO perspectives.

## Conclusions

Advocacy efforts to enhance the role of police in support of HIV programming among key populations have increased over the past 15 years and have focused on police sensitization and training. These efforts have resulted in numerous police training workshops held at national and regional levels. Whilst some of these efforts have resulted in increased police understanding of HIV and harm reduction, some support for HIV and harm reduction programmes at a policy level and the implementation of training curricula [31], rarely have these efforts actually brought local-level police and HIV programmes to resolve long-standing tensions that impact service provision and uptake of services for key populations in high-priority countries.

The proposed model roadmaps a process towards sustained partnership and depending on the context would require the input of some external resources and technical assistance, especially towards reforming national policies and developing police protocols. The resources required would be minor in comparison to the overall budgets of LEAs and health agencies at a national level, highlighting the cost effectiveness of partnership development. The model can be scaled to a local or national level. The series of consultations from which this model is proposed are very much preliminary efforts at each of the national levels and clearly a sustained and supported engagement mechanism is needed. The main focus of the consultations was on enhancing partnerships in the context of creating an enabling environment for scaling up access to the comprehensive package of HIV interventions for people who use drugs [32]; however, many of the workshops discussed the need for partnerships between LEAs and CSOs in the context of HIV among other key populations.

The workshops were designed for 30–40 participants, drawn equally from the LEAs and CSOs active at the country, or sub-national, level. Participants from LEAs were nominated by relevant LEAs, whether at national or sub-national level. Advocacy conducted with high-level police leadership is required to not only conduct consultations of this kind but also to ensure that the right levels of police attend the consultations; this paper recognizes that in many countries this process can be difficult. Representatives of CSOs were identified through nominations received by national networks of CSOs.

While some countries are more advanced in the pursuit of either HIV-related police policy and practice reform or the engagement of CSOs with police, there is a significant amount of work to be done to be able to truly verify sustained partnerships and measure a consequential impact on HIV-risk behaviour and service access and uptake. The analysis emanating from these consultations have enabled the development of the proposed roadmap model which is both scalable and fundable. The roadmap provides a platform for donor partners and host governments to actually cost and evaluate the partnership effort.

The model outlines the need for sustained and ongoing dialogue between LEAs and CSO-led HIV programmes working with key populations, the provision of contextualized yet standardized technical assistance and the development of an increasingly sophisticated partnership agenda. Since these consultations were held, UNODC has developed and begun implementing the “Training Manual for Law Enforcement Officials on HIV Service Provision for People Who Inject Drugs” [33] which acknowledges the need for high-level police to support advocacy efforts in a process articulated in Annex I of the manual. In addition, UNODC has also developed, in partnership with the International Network of People who Use Drugs (INPUD) and LEAHN, the “Practical Guide for Civil Society HIV Service Providers among People who Use Drugs: Improving Cooperation and Interaction with Law Enforcement Officials” [34]. In combination, these documents provide standardized materials for contextualizing and implementing the relevant activities at the country level.

The model builds in a significant component of evaluation which includes the need to first understand at a country context level the current interaction between LEAs and CSOs working on HIV programmes. Second, this model recognizes the need for baseline evaluations that build an understanding of the pre-intervention situation on the ground by conducting knowledge, attitude, behaviour and practice surveys covering a sample size that would give enough power depending on the proposed geographical scope of the roadmap intervention. In addition, the authors recommend the need to measure pre/post measures of police behaviour towards key populations including incidence of arbitrary arrest, violence, the use of bribery and to see if these indicators have decreased at different time points in the proposed two year evaluation. In addition, HIV-risk behaviour should also be surveyed over time to assess the impact of the partnership intervention. Process evaluation of the roadmap, its facilitation and its agenda setting should also be considered. The implementation of such a roadmap would be best accompanied by an implementation science research design.

The need to build collaborative partnerships between LEAs and CSOs in countries where concentrated epidemics of HIV persist among key populations is critical towards ending HIV and would have positive flow on effects to a range of other health and community safety indicators. Despite the opportunities presented through the availability of combination HIV prevention services, it is the access to and uptake of these services which are of equal importance. The pursuit of partnerships between LEAs and CSOs is a goal that should be equally resourced by donor partners and multilaterals from across both the security and health sectors. Resourcing police reform to support HIV-related services has traditionally not been the modus operandi of donors such as The Global Fund for AIDS, Tuberculosis and Malaria. Similarly, police reform in the context of development assistance has rarely been supported with an HIV agenda in mind. A coordinated donor response to police reform is warranted. This paper has highlighted some initial efforts to overcome conflict with collaboration and provides a roadmap to pursue this goal and to sustain this work. It provides policy makers and donor partners with a fundable and scalable model that places the responsibility for creation of an enabling environment firmly within a partnership model on the ground and across the donor environment.
